# Effects of a Novel Tripyrasulfone Herbicide on Key Soil Enzyme Activities in Paddy Rice Soil

**DOI:** 10.3390/plants13223138

**Published:** 2024-11-07

**Authors:** Penglei Sun, He Sun, Shuo Yu, Lei Lian, Tao Jin, Xuegang Peng, Xiangju Li, Weitang Liu, Hengzhi Wang

**Affiliations:** 1 College of Plant Protection, Shandong Agricultural University, Tai’an 271018, China; 2 State Key Laboratory for Biology of Plant Diseases and Insect Pests, Institute of Plant Protection, Chinese Academy of Agricultural Sciences, Beijing 100193, China; pengleisun@163.com (P.S.);; 3 Shandong Province Higher Education Provincial Key Pesticide Toxicology and Application Technology Laboratory, Tai’an 271018, China; 4 Qingdao Kingagroot Crop Science Co., Ltd., Qingdao 266000, China

**Keywords:** ecological risk, QYR301, soil physicochemical properties, microbial enzymes

## Abstract

Weeds significantly impact paddy yields, and herbicides offer a cost-effective, rapid, and efficient solution compared to manual weeding, ensuring agricultural productivity. Tripyrasulfone, a novel 4-hydroxyphenylpyruvate dioxygenase (HPPD) inhibitor developed by Qingdao Kingagroot Chemicals Co., Ltd., has demonstrated high efficacy when applied post-emergence, causing characteristic foliar bleaching in susceptible weed species, distinct from conventional acetolactate synthase, acetyl-CoA carboxylase, and synthetic auxin herbicides. This study investigates the impact of tripyrasulfone on the activity of key soil enzymes (urease (UE), acid phosphatase (ACP), sucrase (SC), catalase (CAT), and dehydrogenase (DHA)) in paddy soils from Jilin Province and Shandong Province. Different doses of tripyrasulfone (0.1, 1.0, and 2.5 mg kg^−1^) were applied, and the enzymatic activities were measured. Results indicated that tripyrasulfone initially inhibited UE and ACP activities before activating them. On the 20th day after treatment, UE activity had returned to control levels, whereas ACP activity remained significantly higher, showing long-lasting activation. SC and CAT activities were inhibited but gradually recovered to control levels. Furthermore, DHA activity was activated with a sustained effect, remaining significantly higher than the control group even 20 days after treatment. Overall, the impact of tripyrasulfone on soil enzyme activities diminished over time, suggesting that tripyrasulfone posed minimal long-term ecological risk to soil health.

## 1. Introduction

Weeds in farmland compete with crops for resources such as nutrients, water, space, and sunlight, while also providing hiding places for pests and crop pathogens, leading to a significant negative impact on arable crop yield and quality [1]. According to statistics, yield losses due to weeds can amount to a maximum estimated loss of 34% of total production annually, resulting in crop losses exceeding USD 100 billion per year [2,3]. In China, there are more than 1400 weed species, resulting in yield reductions causing the annual loss of over 3 million tons of grain [4].

Currently, weed control in farmland mainly includes mechanical weeding, biological weed control, and chemical weed control measures [5]. The application of herbicides with different modes of action is a crucial and widely used measure for chemical weed control in modern agricultural production. These commercial herbicides are highly effective, eliminating 90% to 99% of target weeds, thus offering efficiency, time savings, and labor reduction in weed management practices [6,7,8]. However, the increased use of herbicides has resulted in water and soil pollution, ecological problems, and negative impacts on the environment, affecting the health of animals and humans. Applying herbicides to crops can lead to adverse effects, including qualitative and quantitative changes in soil microbial populations and alterations in the activity of soil enzymes. These effects are strongly linked to the concentration and persistence of the herbicide, its physicochemical properties and behavior, as well as the physicochemical parameters of the soil [9,10,11,12,13].

Soil enzymes are natural active proteins secreted by living microorganisms, plants, and soil fauna, or released during the decomposition of dead organisms [14,15]. They play a crucial role in catalyzing reactions necessary for organic matter decomposition, nutrient release, energy transfer, and maintaining environmental quality [16]. The activity of soil enzymes is considered an integrative bioindicator, providing valuable information about soil health and fertility [17,18]. Hence, soil enzyme activities are often used to monitor the impacts of soil management, agricultural practices, and environmental contamination on soil health [19]. Soil enzyme activities respond rapidly to environmental changes, effectively reflecting the extent of soil contamination, energy metabolism, pollutant degradation, and nutrient transformation processes [20]. Examples of such enzymes include urease (UE), acid phosphatase (ACP), sucrase (SC), catalase (CAT), and dehydrogenase (DHA) [15,21,22,23,24,25]. UE in soil catalyzes the hydrolysis of urea, producing carbonate and ammonia. Its activity is positively correlated with the content of organic matter, microbial population, available nitrogen, and total nitrogen in the soil, thereby reflecting the soil’s nitrogen status [15]. Studies on soybean field soils have found that alachlor, propaquizafop, and imazethapyr can stimulate urease activity [26]. ACP in soil can catalyze the mineralization of organic phosphorus under acidic conditions. Its activity directly influences the decomposition, transformation, and bioavailability of organic phosphorus compounds, serving as an indicator of the soil’s phosphorus bio-transformation intensity and direction [15]. Continuous application of 432 mg of a.e. glyphosate (a non-selective herbicide that inhibits 5-enolpyruvylshikimate-3-phosphate synthase) has been reported to increase the acidity of clay soils and enhance ACP activity [27]. SC in soil catalyzes the hydrolysis of sucrose into monosaccharides, which organisms can then utilize. The products of this enzymatic reaction are closely related to soil organic matter content, soil respiration intensity, and microbial population, making SC an important indicator of soil fertility [15,28]. QYM201, which inhibits 4-hydroxyphenylpyruvate dioxygenase (HPPD), has been reported to initially reduce SC activity in brown loam soil, with the inhibitory effect strengthening as the concentration increases to 0.1, 1.0, and 5.0 mg kg^−1^ [25]. CAT in soil is a reductase that effectively mitigates the toxic effects of hydrogen peroxide (H_2_O_2_) produced during soil metabolism [29]. Research has indicated that a concentration of 1 mg kg^−1^ of QYM201 significantly enhances CAT activity in brown loam soil. Additionally, the correlation between CAT activity and herbicides like bensulfuron-methyl and chlorsulfuron-methyl has been reported [25]. DHA activity reflects the number of active microorganisms in the soil system and their ability to degrade organic matter, serving as an indicator of the soil’s microbial degradation performance [19]. The results of Du et al. (2018) showed that dehydrogenase (DHA) activity in brunisolic soil initially decreased with increasing concentrations of mesotrione (0.1 to 5 mg/kg), but then recovered over time [24]. Additionally, Pose-Juan et al. (2015) investigated the effects of 50 mg/kg of mesotrione in sandy loam soil, finding that it increased soil microbial biomass while reducing dehydrogenase activity [30]. These indicators are widely used both domestically and internationally to study soil responses to pesticide residues.

**Figure 1:**
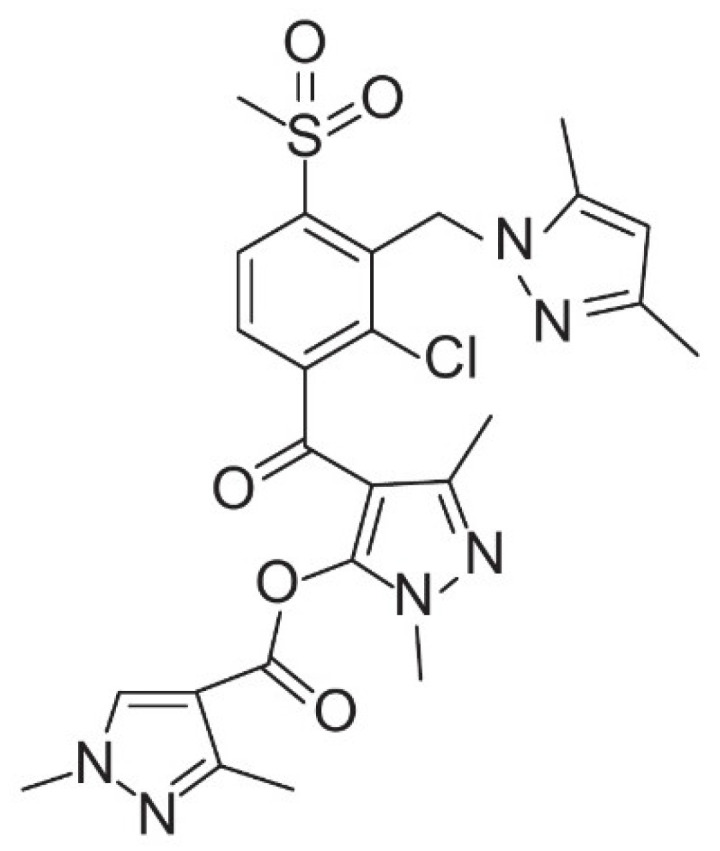
The structural formula of tripyrasulfone.

Tripyrasulfone (97.8% purity, CAS: 1911613-97-2), with the IUPAC name [4-[2-chloro-3-[(3,5-dimethylpyrazol-1-yl)methyl]-4-methylsulfonylbenzoyl]-2,5-dimethylpyrazol-3-yl] 1,3-dimethylpyrazole-4-carboxylate and code name QYR301, is a novel HPPD inhibitor herbicide developed in 2011 with independent intellectual property rights by Qingdao Kingagroot Chemical Compound Co., Ltd. (Qingdao, China) in collaboration with Shandong Agricultural University [6,31]. The structural formula is shown in Figure 1.

Due to the recent development of tripyrasulfone, which was commercialized in China in 2020, research on its effects has remained limited. Our previous studies demonstrated that tripyrasulfone was highly safe for rice, effectively controlled the major paddy weed *Echinochloa crus-galli* (L.) P. Beauv., and exhibited low toxicity to paddy field environments and non-target organisms [6,31,32,33,34]. However, the impact of tripyrasulfone on soil enzyme activities has not yet been investigated. Therefore, this study aims to elucidate its effects on the activities of five soil enzymes (UE, ACP, SC, CAT, and DHA) in two different soil types. It is hypothesized that the application of tripyrasulfone may differentially influence these enzyme activities, potentially resulting in significant changes to soil health and nutrient cycling due to its new chemical structure and mode of action as a newly developed herbicide.

## 2. Results

### 2.1. Effect of Tripyrasulfone on UE Activity in Paddy Soils

**Figure 2:**
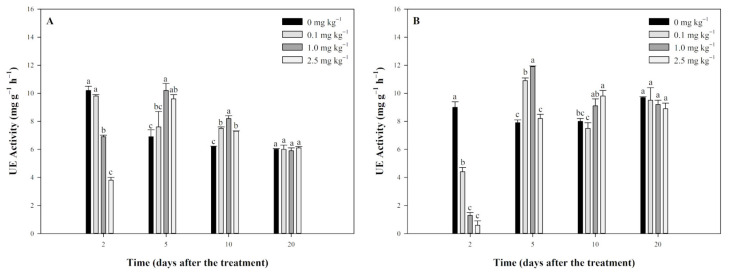
Effect of tripyrasulfone on urease (UE) activity in different soils. Data represent the mean plus or minus standard deviation (*n* = 3). Different letters indicate significant differences at the *p* < 0.05 level according to Fisher’s protected LSD test. (**A**): JL-SY soil; (**B**): SD-LY soil.

The effects of tripyrasulfone on UE activity in JL-SY and SD-LY paddy soils are shown in Figure 2. The results of JL-SY soil indicated that the UE activity of treated groups was initially inhibited and then activated by tripyrasulfone. On the 2nd day, UE activity in all treated groups was lower than the control, with the degree of inhibition increasing with higher concentrations of tripyrasulfone, and significant inhibition was observed in the 1.0 mg kg^−1^ and 2.5 mg kg^−1^ treatment groups. On the 5th day, UE activity in all treated groups was higher than the control, with significant increases in the 1.0 mg kg^−1^ and 2.5 mg kg^−1^ groups. On the 10th day, all treated groups showed significantly higher UE activity than the control. By the 20th day, UE activity had returned to normal levels comparable to the control in all treated groups (Figure 2A).

The effects of tripyrasulfone on SD-LY soil were similar to those observed in JL-SY soil (Figure 2B). On the 2nd day, UE activity was significantly lower in all treated groups compared to the control, with higher concentrations showing more inhibition. On the 5th day, UE activity was higher than the control in all treated groups, with significant levels in the 1.0 mg kg^−1^ and 2.5 mg kg^−1^ groups. On the 10th day, UE activity in the 0.1 mg kg^−1^ group returned to control levels, while the 1.0 mg kg^−1^ and 2.5 mg kg^−1^ groups remained higher. By the 20th day, UE activity in all groups returned to control levels.

### 2.2. Effect of Tripyrasulfone on SC Activity in Paddy Soil

**Figure 3:**
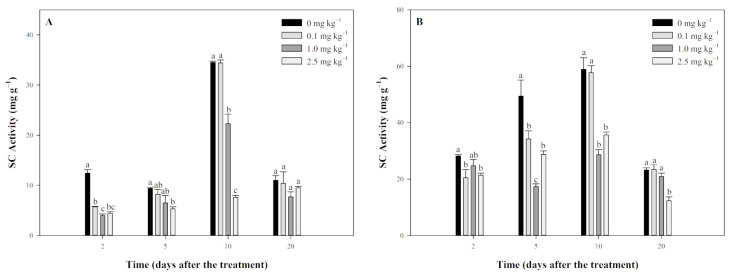
Effect of tripyrasulfone on sucrase (SC) activity in different soils. Data represent the mean plus or minus standard deviation (*n* = 3). Different letters indicate significant differences at the *p* < 0.05 level according to Fisher’s protected LSD test. (**A**): JL-SY soil; (**B**): SD-LY soil.

Figure 3 shows the effect of tripyrasulfone on SC activity in JL-SY and SD-LY paddy soils. In JL-SY soil (Figure 3A), tripyrasulfone initially inhibited SC activity. On the 2nd day, SC activity in all treatment groups was significantly lower than in the control. On the 5th day, this inhibition persisted, with significant effects observed in the 2.0 mg kg^−1^ group. On the 10th day, SC activity in the 0.1 mg kg^−1^ group returned to control levels, whereas the 1.0 mg kg^−1^ and 2.0 mg kg^−1^ groups remained significantly inhibited. By the 20th day, SC activity in all groups returned to control levels.

In SD-LY soil (Figure 3B), a similar inhibitory trend was observed. On the 2nd day, SC activity in all treated groups was significantly lower than in the control, with significant inhibition in the 0.1 mg kg^−1^ and 2.5 mg kg^−1^ groups. On the 5th day, the inhibition intensified, with all groups showing significantly lower SC activity. On the 10th day, SC activity in the 0.1 mg kg^−1^ group returned to control levels, while the 0.1 mg kg^−1^ and 2.5 mg kg^−1^ groups remained inhibited. By the 20th day, SC activity in the 0.1 mg kg^−1^ and 1.0 mg kg^−1^ groups returned to control levels, but the 2.5 mg kg^−1^ group remained significantly inhibited.

### 2.3. Effect of Tripyrasulfone on CAT Activity in Paddy Soil

**Figure 4:**
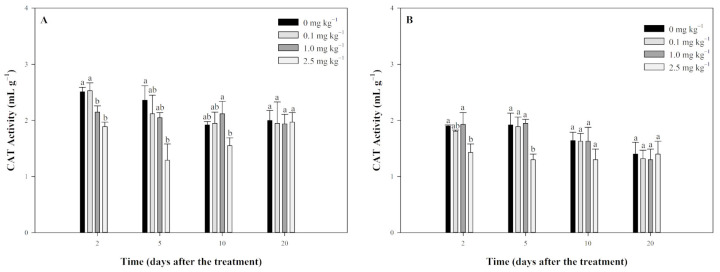
Effect of tripyrasulfone on catalase (CAT) activity in different soils. Data represent the mean plus or minus standard deviation (*n* = 3). Different letters indicate significant differences at the *p* < 0.05 level according to Fisher’s protected LSD test. (**A**): JL-SY soil; (**B**): SD-LY soil.

As shown in Figure 4A, tripyrasulfone generally inhibited CAT activity in JL-SY soils. The 0.1 mg kg^−1^ treatment group showed no significant difference from the control throughout the experiment. On the 2nd day, CAT activity in the 1.0 mg kg^−1^ and 2.5 mg kg^−1^ treatment groups was significantly lower than in the control. By the 5th day, both the 1.0 mg kg^−1^ and 2.5 mg kg^−1^ groups remained inhibited, with a significant difference observed only in the 2.5 mg kg^−1^ group. On the 10th day, only the 2.5 mg kg^−1^ group had significantly lower CAT activity compared to the control. By the 20th day, CAT activity in all treatment groups had returned to control levels.

In SD-LY soil (Figure 4B), the results indicated that tripyrasulfone also inhibited CAT activity, but the effect was weaker. Only the high-dose 2.5 mg kg^−1^ group showed significant inhibition on days 2 and 5, with CAT activity returning to control levels by day 20. The other treatment groups showed no significant difference from the control throughout the experiment.

### 2.4. Effect of Tripyrasulfone on DHA Activity in Paddy Soil

**Figure 5:**
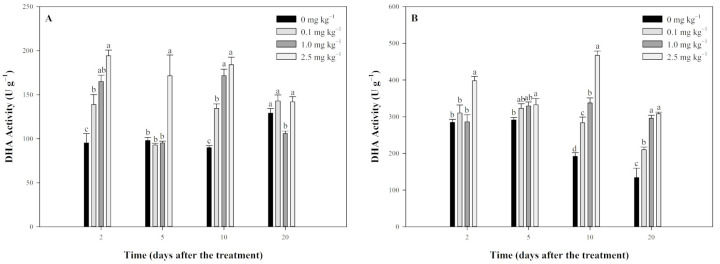
Effect of tripyrasulfone on dehydrogenases (DHA) activity in different soils. Data represent the mean plus or minus standard deviation (*n* = 3). Different letters indicate significant differences at the *p* < 0.05 level according to Fisher’s protected LSD test. (**A**): JL-SY soil; (**B**): SD-LY soil.

DHA activity in JL-SY soils increased after tripyrasulfone treatment (Figure 5A), indicating that tripyrasulfone generally activated DHA activity. Specifically, on the 2nd day, all treatment groups exhibited significantly higher DHA activity compared to the control. By the 5th day, the 0.1 mg kg^−1^ and 1.0 mg kg^−1^ groups returned to control levels, while the 2.5 mg kg^−1^ group maintained significantly higher activity. On the 10th day, all treatment groups showed significantly higher DHA activity than the control. By the 20th day, DHA activity in the 0.1 mg kg^−1^ and 2.5 mg kg^−1^ groups returned to control levels, while the 1.0 mg kg^−1^ group showed significantly lower activity compared to the control.

The result of DHA activity in SD-LY soil showed that tripyrasulfone also activated DHA activity, with a more sustained effect (Figure 5B). On the 2nd day, the 0.1 mg kg^−1^ and 1.0 mg kg^−1^ groups showed no significant difference from the control, but the 2.5 mg kg^−1^ group had significantly higher DHA activity. By the 5th day, all treatment groups exhibited higher DHA activity compared to the control, with only the 2.5 mg kg^−1^ group showing a significant difference. On the 10th and 20th days, all treatment groups had significantly higher DHA activity than the control.

### 2.5. Effect of Tripyrasulfone on ACP Activity in Paddy Soil

**Figure 6:**
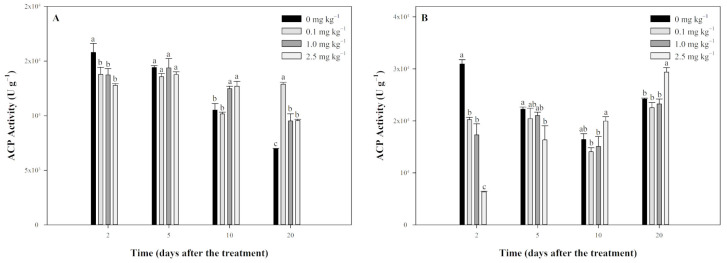
Effect of tripyrasulfone on acid phosphatase (ACP) activity in different soils. Data represent the mean plus or minus standard deviation (*n* = 3). Different letters indicate significant differences at the *p* < 0.05 level according to Fisher’s protected LSD test. (**A**): JL-SY soil; (**B**): SD-LY soil.

The effect of tripyrasulfone on ACP activity in JL-SY and SD-LY paddy soils was similar to UE activity, showing an initial inhibition followed by activation (Figure 6). In JL-SY soil (Figure 6A), on the 2nd day, tripyrasulfone significantly inhibited ACP activity in all treatment groups compared to the control. By the 5th day, ACP activity in all treatment groups returned to control levels. On the 10th day, ACP activity was activated by tripyrasulfone, with the 0.1 mg kg^−1^ group showing no significant difference from the control, while the 1.0 mg kg^−1^ and 2.5 mg kg^−1^ groups had significantly higher ACP activity. By the 20th day, the activation effect persisted, with all treatment groups showing significantly higher ACP activity than the control.

In SD-LY soil (Figure 6B), on the 2nd day, all treatment groups exhibited significantly lower ACP activity compared to the control. By the 5th day, ACP activity in the 0.1 mg kg^−1^ and 1.0 mg kg^−1^ groups returned to control levels, while the 2.5 mg kg^−1^ group still showed significantly lower activity. On the 10th and 20th days, ACP activity in the 0.1 mg kg^−1^ and 1.0 mg kg^−1^ groups returned to control levels, whereas the 2.5 mg kg^−1^ group showed significantly higher ACP activity, indicating activation.

## 3. Discussion

As a post-emergence herbicide, tripyrasulfone, when sprayed in direct-seeded rice fields where both rice and weeds are at an early growth stage, can result in some of the herbicide solution being directly applied to the soil surface or dripping down from the leaves onto the soil. Once in the soil, the herbicide or its metabolites may persist, potentially impacting soil enzyme activities and thereby affecting normal biochemical reactions in the soil ecosystem, posing potential hazards to soil environments [35,36,37,38]. Therefore, this study measured the impact of tripyrasulfone at doses of 0.1, 1.0, and 2.5 mg kg^−1^ on the activities of UE, SC, CAT, DHA, and ACP in paddy soils collected from Songyuan city, Jilin Province, and Linyi city, Shandong Province. These five enzymes are good indicators of soil fertility, energy metabolism, pollutant degradation, and nutrient transformation capabilities.

The results of this study indicated that tripyrasulfone initially inhibited and then activated soil UE activity, similar to the effects of glyphosate on UE activity in cotton field soil [39]. Additionally, others reported that the same concentration of glyphosate led to differing responses of this enzyme across soils with varying properties [39]. Our research found that two days after tripyrasulfone treatment, UE activity at all doses was significantly lower than the control, with UE activity in SD-LY soil showing more pronounced inhibition compared to JL-SY. This difference may be attributed to variations in the native microbial abundance and the physicochemical properties of the soils. However, on the 20th day after tripyrasulfone treatment, there were no differences in UE activity across different tripyrasulfone dose treatments compared to the control, suggesting that tripyrasulfone does not have any potential adverse effects on UE activity in either soil. Tripyrasulfone also exhibited a similar inhibitory-then-activating effect on soil ACP activity. It has been reported that herbicides can directly affect soil enzyme activity through interaction with soil enzymes or indirectly affect it by interacting with soil microorganisms [40]. When tripyrasulfone entered the soil, it initially inhibited the activity of UE and ACP by directly interacting with the enzymes. Over time, as tripyrasulfone degraded (with a half-life in soil of 4.3 to 8.0 days in field conditions) [32], it provided nitrogen and carbon sources for soil microorganisms, which enhanced the activity of UE and ACP. Based on our experimental results, although the initial level of ACP activity in SD-LY soil differed from that in JL-SY, both soils exhibited a consistent response of ACP activity to tripyrasulfone. We hypothesize that the initial levels of ACP activity in different soils do not significantly affect the impact of tripyrasulfone on ACP activity. By the fifth day after treatment, ACP activity in both soils returned to initial levels, indicating that tripyrasulfone does not have detrimental effects on ACP activity in different soils.

Soil SC is an important hydrolytic enzyme that indicates soil biological activity and reflects soil fertility. This study found that soil SC activity was strongly inhibited by tripyrasulfone, with the inhibition increasing with higher concentrations. In the later stages, the high-dose treatment groups in SD-LY soil did not recover to control levels, which is consistent with the effect of the HPPD inhibitor herbicide isoxaflutole on SC activity in wheat field soil [25]. The differences in SC activity between the two soils may be related to variations in soil properties. Specifically, the initial SC activity in SD-LY soil was generally higher than in JL-SY soil, possibly due to the higher organic matter content in Shandong soil. Generally, higher soil fertility correlates with increased SC activity [25]. Therefore, we suspect that the SC activity in SD-LY soil may require a longer time to recover.

Soil CAT is widely distributed in soil and facilitates the decomposition of H_2_O_2_, preventing the toxic effects of H_2_O_2_ produced during metabolism. This study found that soil CAT was insensitive to tripyrasulfone, with only high doses causing inhibition of its activity, which returned to control levels in the later stages of the experiment. The research has shown that CAT activity in soil exhibited a slow decline following low concentrations of chlorimuron-ethyl treatment, while high concentrations resulted in a significant decrease [41]. This finding is similar to the significant inhibition of CAT observed only at high doses of tripyrasulfone in this study. Additionally, other authors have reported a phenomenon where chlorpyrifos initially inhibits and then allows recovery of CAT activity in soil. This observation aligns with our results, indicating that tripyrasulfone does not have adverse effects on CAT activity in soil [42].

This study also found that tripyrasulfone had a long-lasting activating effect on soil DHA activity, with some treatments not returning to control levels even after 20 days. This could be because soil microorganisms producing DHA quickly adapted to the tripyrasulfone-contaminated microenvironment, potentially using tripyrasulfone as a carbon and energy source for rapid growth, thus enhancing DHA activity. As low concentrations of tripyrasulfone were depleted, the reduced availability of carbon sources led to a slowdown in microbial growth, causing DHA activity to return to control levels. However, high concentrations provided a longer-term carbon and energy source, maintaining the activation of soil DHA for an extended period. These results are similar to those found by Ma et al. in their study on Chlorimuron-ethyl [41].

In our previous study on the toxicity and enzymatic activity in earthworms exposed to 2.5 mg kg^−1^ tripyrasulfone, we observed that CAT activity was inhibited after 21 days of treatment, and tripyrasulfone induced excessive ROS production, oxidative stress, lipid membrane peroxidation, and ultimately DNA damage in the earthworms [34]. In contrast, the trend of CAT activity in soil following 2.5 mg kg^−1^ tripyrasulfone treatment differed from that observed in earthworms. Tripyrasulfone did not cause sustained inhibition of CAT activity in soil; therefore, the residual tripyrasulfone in soil may have a lesser impact on soil than on earthworms.

## 4. Materials and Methods

### 4.1. Herbicide

Tripyrasulfone (97.8% purity) was provided by Qingdao Kingagroot Chemical Compound Co., Ltd. (Qingdao, China).

### 4.2. Soil Sampling

The soil samples used in this research were collected from rice fields in Ningjiang district, Songyuan city, Jilin province (JL-SY, 45°11′23.39″ N, 124°50′32.62″ E), and Hedong district, Linyi city, Shandong province (SD-LY, 35°11′6.11″ N, 118°30′37.16″ E). Neither site had been treated with tripyrasulfone before collection. Using a soil sampler (sampling depth of 10 cm), samples were obtained through a 5-point sampling method, yielding quantities between 20 and 25 kg. Gravel, plant residues, and other debris were removed. The soil samples were uniformly mixed, air-dried, and passed through a 20-mesh sieve (<0.85 mm). The soil samples were adjusted to 60% maximum water-holding capacity (WHC) of the field capacity to stabilize soil microbial levels and incubated in the dark at 25 °C for 14 days. The basic physicochemical characteristics of the two soils are shown in Table 1 [43].

### 4.3. Treatment of Soil Samples with Tripyrasulfone

The initial field residue levels of tripyrasulfone range from 0.01 to 1.0 mg kg^−1^ as our research reported [32]. Therefore, this study established treatment groups with nominal tripyrasulfone concentrations of 0.1, 1.0, and 2.5 mg·kg^−1^. Tripyrasulfone-acetone solutions were prepared using 1 mL of acetone as the solvent, with concentrations of 5, 50, and 125 mg·L⁻^1^. Each treatment involved mixing 10 g of dry soil with the corresponding concentration of the tripyrasulfone-acetone solution, while the control group (0 mg·kg^−1^) received an equal volume of acetone added to 10 g of dry soil. The mixtures were then allowed to fully evaporate in a fume hood. Finally, the treated soil samples were thoroughly mixed with 490 g of soil at 60% WHC, resulting in soil samples with tripyrasulfone concentrations of 0, 0.1, 1.0, and 2.5 mg·kg^−1^. All samples were then incubated in the dark at a constant temperature of 25 °C. An acetone-only treatment served as the control group, and each treatment was replicated three times. Samples were collected at 2, 5, 10, and 20 days after treatment. During the incubation period, sterile water was used to maintain soil moisture at 60% of the WHC by adding it to the soil surface every 2 days.

### 4.4. Soil UE Activity Assay

Soil UE activity was measured using a modified phenol-sodium hypochlorite colorimetric method [44,45]. To assess UE activity, 10 g of air-dried contaminated soil was mixed with 2.0 mL of toluene and left to stand at 25 °C for 15 min. Then, 10 mL of 10% urea solution and 20 mL of citrate buffer (pH 6.7) were added and mixed. The mixture was incubated at 37 °C for 3 h. After incubation, the volume was adjusted to 100 mL with 38 °C deionized water, thoroughly mixed, and allowed to stand undisturbed for 1 min. The toluene was then removed, and the mixture was filtered. A 1.0 mL aliquot of the filtrate was mixed with 19 mL of deionized water, 4.0 mL of sodium phenol solution, and 3.0 mL of NaClO solution. After mixing, the solution was left to stand for 20 min, and the volume was adjusted to 50 mL with deionized water. The optical density at 578 nm (OD_578_) was measured using a UV–Vis spectrophotometer. Controls without substrate (deionized water replacing urea solution) and without soil were also measured. For the standard curve, 1.0, 3.0, 5.0, 7.0, 9.0, 11.0, and 13.0 mL of 0.01 mg mL^−1^ nitrogen standard solution were added to 50 mL volumetric flasks, filled to 20 mL with deionized water, then mixed with 4.0 mL of sodium phenol solution and 3.0 mL of NaClO solution. After a 20 min color development period, the volume was adjusted to 50 mL with deionized water, and OD_578_ was measured. The standard curve was plotted using NH_4_^+^-N concentration as the X-axis and OD_578_ as the Y-axis.

Soil UE activity (U, mg g^−1^ h^−1^) was expressed as the amount of NH_4_^+^-N produced per gram of soil per hour. The activity was calculated using the formula:

$$U=\frac{V\times C\times \left(X_{1}-X_{2}-X_{3}\right)}{m\times t}$$

where X_1_, X_2_, and X_3_ represent the NH_4_-N concentrations in the sample, soil-free control, and substrate-free control, respectively; V is the ratio of the volume of the sample solution to the volume of the measured sample; C is the coefficient converting NH_4_-N concentration from the standard curve to mass; m is the ratio of the enzyme activity unit to the soil weight; and t is the incubation time.

### 4.5. Soil SC Activity Assay

The soil SC activity assay was performed according to the reported method [46]. A mass of 5 g of air-dried contaminated soil, sieved to less than 1 mm, was placed in a 50 mL Erlenmeyer flask, followed by the addition of 15 mL sucrose solution, 5 mL 0.2 mol L^−1^ phosphate buffered saline (PBS) buffer (pH 5.5), and 5 drops of toluene. The mixture was thoroughly mixed and incubated at 37 °C for 24 h. After incubation, the mixture was quickly filtered. A 1 mL aliquot of the filtrate and 3 mL of 3,5-dinitrosalicylic acid solution were transferred to a 50 mL volumetric flask and heated in boiling water for 5 min. The flask was then cooled under running water for 3 min. The volume was adjusted with deionized water, and the optical density at 508 nm (OD_508_) was measured using a spectrophotometer. Each soil sample included a substrate-free control, and the entire experiment included a soil-free control. Simultaneously with sample analysis, 0, 1, 2, 3, 4, 5, 6, and 7 mL of glucose working solution (0.5 mg mL^−1^) were each transferred into 50 mL volumetric flasks. These solutions underwent color development using the same method as the SC activity assay. After colorimetric analysis, a standard curve was plotted with absorbance as the Y-axis and glucose concentration as the X-axis.

SC activity (U, mg g^−1^) was expressed as the mass of glucose per gram of soil after 24 h, calculated using the formula:

$$U=\frac{a\times V\times N}{m}$$

where a is the glucose concentration obtained from the standard curve, V is the volume of the colored solution, n is the dilution factor, and m is the mass of the dry soil.

### 4.6. Soil CAT Activity Assay

Soil CAT activity was measured by back-titrating the residual H_2_O_2_ with KMnO_4_, with slight modifications [47,48]. Specifically, 2 g of air-dried contaminated soil was placed in a 100 mL Erlenmeyer flask, followed by the addition of 40 mL deionized water and 5 mL of 0.3% H_2_O_2_ solution. The control was also prepared by adding 40 mL of deionized water and 5 mL of 0.3% H_2_O_2_ solution to an Erlenmeyer flask without soil. Both flasks were shaken for 20 min. Then, 5 mL of 1.5 mol L^−1^ sulfuric acid was added to stabilize the unreacted H_2_O_2_. The suspension in the flask was filtered using slow filter paper. A 25 mL aliquot of the filtrate was then titrated with 0.02 mol L^−1^ KMnO_4_ solution to a light pink endpoint.

CAT activity (U, mL g^−1^) was expressed as the volume of 0.02 mol L^−1^ KMnO_4_ solution consumed per gram of soil after 20 min. The activity was calculated using the formula $U=\frac{\left(A\;-\;B\right)}{m}$, where A is the volume of KMnO_4_ solution (in mL) used to titrate 25 mL of the original H_2_O_2_ solution, B is the volume of KMnO_4_ solution (in mL) used to titrate the soil filtrate, and m is the mass of the air-dried soil sample.

### 4.7. Soil DHA Activity Assay

Soil DHA activity was measured using the SDHA-1-G kit from Suzhou Keming Biotechnology Co., Ltd., Suzhou, China (http://www.cominbio.com/a/shijihe/shenghuashiji/turangxilie/2014/1113/575.html, accessed on 21 January 2021).

### 4.8. Soil ACP Activity Assay

Soil ACP activity was measured using the BC0145 kit from Beijing Solarbio Science & Technology Co., Ltd., Beijing, China (https://www.solarbio.com/goodsInfo?id=9122, accessed on 21 January 2021).

### 4.9. Statistical Analysis

All data were analyzed using SPSS 22.0 software. The significance of differences between samples and the control group was determined using the LSD test in one-way ANOVA. All graphs were created using SigmaPlot 12.5.

## 5. Conclusions

This study investigated the effects of the novel compound tripyrasulfone on soil enzyme activities in two types of soil. Following treatment with tripyrasulfone, soil UE and ACP activities initially showed inhibition followed by activation, while soil SC and CAT activities were inhibited. In contrast, soil DHA activity was activated. Overall, the impact of tripyrasulfone on soil enzyme activities diminished over time, gradually returning to control levels by the end of the experiment. Although the two types of soil in this study exhibited differences in physicochemical properties, microbial abundance, and nutrient content, the impact of tripyrasulfone on the activities of the five enzymes showed a consistent trend across both soils. As a novel HPPD inhibitor, the effects of tripyrasulfone on soil enzyme activities in paddy fields were found to be temporary and did not pose a threat to soil health. The diminishing effects on soil enzyme activity towards the end of the experiment, with levels returning to control, suggest that tripyrasulfone poses relatively minor ecological hazards when introduced into the soil environment.

## Figures and Tables

**Table 1:** The basic physicochemical characteristics of the experimental soils.

Physicochemical Characteristics	Soils	
	JL-SY	SD-LY
pH	5.82	7.08
Organic matter (g kg^−1^)	37.37	43.70
N (mg kg^−1^)	116.38	69.25
P (mg kg^−1^)	58.78	195.07
K (mg kg^−1^)	248.95	134.05

Note: N, total nitrogen; P, available phosphorus; K, available potassium.

## Data Availability

Data are contained within the article.
